# Grief during the COVID-19 pandemic: A cross-sectional online survey in university students

**DOI:** 10.1192/j.eurpsy.2022.678

**Published:** 2022-09-01

**Authors:** M.J. Soares, D. Pereira, A.P. Amaral, J. Azevedo, S. Bos, A.T. Pereira, N. Madeira, A. Macedo

**Affiliations:** 1 Faculty of Medicine of University of Coimbra, Institute Of Psychological Medicine, Coimbra, Portugal; 2 Centro Hospitalar e Universitário de Coimbra, Department Of Psychiatry, Coimbra, Portugal; 3 Polytechnical Institute of Coimbra, Coimbra Health School, Coimbra, Portugal

**Keywords:** grief, students, Covid-19

## Abstract

**Introduction:**

Almost 5 million people worldwide have lost their lives due to SARS-CoV-2 (source: WHO coronavirus (COVID-19) dashboard, data of 1.10.2021; https://covid19.who.int/) and therefore, globally, there is an increase of people in grief due to the death of a significant other.

**Objectives:**

To study psychological correlates of grief during the COVID-19 pandemic.

**Methods:**

591 university students, with a mean age of 23.84±7.95 years (range 18-65 years; 76.8% women; 91.2% Portuguese) completed an online questionnaire during the second COVID-19 confinement (from 15.02 to 13.03.2021), with sociodemographic questions, the Pandemic Stress Index, the Mental Health Inventory, Insomnia Scale, questions on physical/ psychological health, and social isolation.

**Results:**

Students bereaving the death of a significant other (n=93, 15.7%; n=25, 26.9% reported cause was SARS-CoV-2; time since death: < 3 months to 1-year), compared to those who did not (n= 498; 84.3%), described poorer psychological health, higher psychological distress (depression, anxiety, lack of control) and sleep difficulties, higher levels of stress (higher impact of COVID pandemic in daily life, and higher behavior changes in response to COVID-19) and more social isolation.

**Conclusions:**

COVID-19 pandemic-related stress is a source of additional stress for bereaved students. Grief is also associated with social isolation, poor mental health (depression, anxiety, lack of control) and sleep difficulties. Screening efforts, guidance, and counseling from professionals of mental health care, primary health care, and universities health care services during and after the COVID-19 pandemic could be extremely beneficial for bereaved students, particularly for those at higher risk of developing prolonged grief disorder.

**Disclosure:**

No significant relationships.

